# EXAMINING THE DIMENSIONALITY OF FUNCTIONAL DISABILITY MEASURES FOR OLDER ADULTS

**DOI:** 10.1093/geroni/igae098.2607

**Published:** 2024-12-31

**Authors:** Rachel Upton, Kristen Robinson

**Affiliations:** ACL/HHS, Washington, District of Columbia, United States; Administration for Community Living, Washington, District of Columbia, United States

## Abstract

The National Survey of Older Americans Act Participants (NSOAAP) is an annual survey that evaluates programs on aging funded by Title III of the Older Americans Act (OAA). This session will discuss a cross-sectional study that used item factor analysis (IFA) and 2021 NSOAAP data to examine the dimensionality of functional disability measures used to classify older adults potentially at-risk for nursing home placement. Functional disabilities comprised limitations in activities of daily living (ADLs) and instrumental activities of daily living (IADLs). ADLs were defined as basic physical tasks required for independently caring for oneself (e.g., eating, bathing) (Fleishman, Spector, & Altman, 2002). IADLs similarly involved independent living activities such as going outside or performing housework. Research suggests that older adults can be classified as potentially at-risk for institutionalization if they display three or more functional disabilities (Tillery, 2021). However, no studies have examined whether current ADL-/IADL-limitation measures used to identify potentially at-risk older adults were unidimensional, such that these measures were accurately scored as reflecting a single construct versus being broken down into multiple, multidimensional subscales (Reise et al., 2010). Analyses focused on caregivers who reported ADL-/IADL-limitations for their care recipients and received OAA Title III caregiver support services within the past year. IFA models indicated that the ADL-limitation measure exhibited a unidimensional factor structure. Bifactor models suggested that the IADL-limitation measure was not unidimensional, with a significant portion of item variance attributed to additional subdomains or subscale(s). Overall, findings reveal the importance of assessing dimensionality for functional disability measures.

